# Computed Tomography Imaging in Lemmel Syndrome: A Report of Two Cases

**DOI:** 10.25259/JCIS-17-2019

**Published:** 2019-05-24

**Authors:** Giulia Frauenfelder, Annamaria Maraziti, Vincenzo Ciccone, Giuliano Maraziti, Oliviero Caleo, Francesco Giurazza, Bruno Beomonte Zobel, Mattia Carbone

**Affiliations:** 1 Departments of Radiology, Università Campus-Bio Medico di Roma, Via A. del Portillo, Rome; 2 Departments of Radiology, San Giovanni e Ruggi D’Aragona Hospital, Ospedale, Via San Leonardo, Salerno; 3 Department of Interventional Radiology, AORN Cardarelli Hospital, Via Antonio Cardarelli, Naples, Napoli, Italy.

**Keywords:** Periampullary duodenal diverticula, Lemmel syndrome, Computed tomography, Common bile duct, Abdominal pain

## Abstract

Lemmel syndrome is a rare and misdiagnosed cause of acute abdominal pain due to a juxtapapillary duodenal diverticulum causing mechanical obstruction of the common bile duct. Frequently, patients suffering from Lemmel syndrome have a history of recurrent access to the emergency room for acute abdominal pain referable to a biliopancreatic obstruction, in the absence of lithiasis nuclei or solid lesions at radiological examinations. Ultrasonography (US) may be helpful in evaluation of upstream dilatation of extra-/intra-hepatic biliary duct, but computed tomography (CT) is the reference imaging modality for the diagnosis of periampullary duodenal diverticula compressing the intrapancreatic portion of the common bile duct. Recognition of this entity is crucial for targeted, timely therapy avoiding mismanagement and therapeutic delay. The aim of this paper is to report CT imaging findings and our experience in two patients affected by Lemmel syndrome.

## INTRODUCTION

Lemmel syndrome, first described by Lemmel in 1934,^[1]^ is defined as obstructive jaundice caused by periampullary duodenal diverticulum (PAD) in the absence of choledocholithiasis or neoplasm. PAD incidence increases with age, observed in around 10–20% of patients undergoing endoscopic retrograde cholangiopancreatography (ERCP), with no gender predilection. PAD is acquired extraluminal outpouching of the duodenal wall through “*locus* *minoris* resistance” located within 2–3 cm to ampulla of Vater.^[2]^ Even if usually asymptomatic, PAD can cause acute abdominal pain due to an extrinsic obstruction of common bile duct or pancreatic duct, miming a biliopancreatic colic. While acute abdomen resulting from common intra-abdominal pathology has been extensively covered in the medical literature as well as the role of cross-sectional imaging in depicting acute or subacute abdominal conditions, the role of imaging in the diagnosis of symptomatic duodenal diverticula is an underreported entity,^[3]^ mainly due to the absence of specific pathognomonic symptoms and signs.

**Figure 1:**
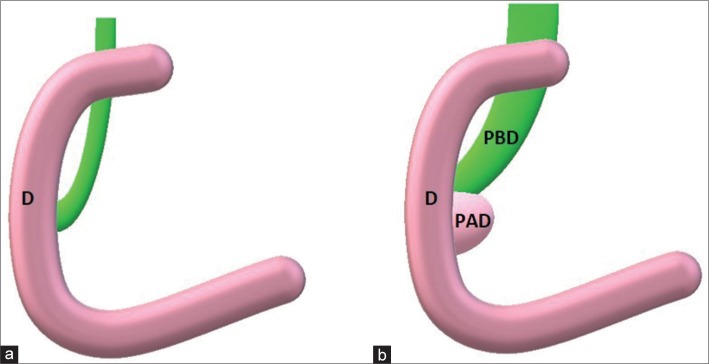
Schematic representation of periampullary duodenal diverticulum. (a) Principal bile duct normally between 4 and 8 mm in maximum diameter. (b) When a periampullary duodenal diverticulum is present, its extrinsic compression could enlarge periampullary duodenal diverticulum till obstructive jaundice. (PBD = Principal bile duct; PAD = Periampullary duodenal diverticulum; d = duodenum).

Lemmel’s syndrome could be challenging to diagnose, but recognize this condition is crucial to avoid wrong treatment. The first step in diagnosis is the identification of PAD [Figure 1].

**Figure 2:**
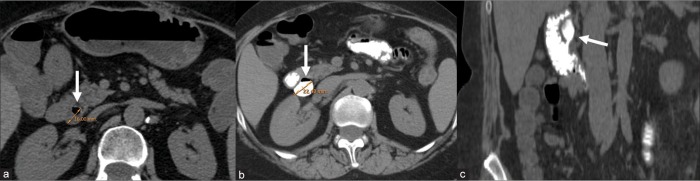
A 64-year-old woman with a history of recurrent postprandial epigastric pain. After US examination, which demonstrated common bile duct dilatation in the absence of lithiasis nuclei, the patient underwent unenhanced computed tomography (a), which demonstrated a periampullary duodenal diverticulum (white arrow) with a strict neck. Oral contrast was administered to better identify diverticulum, which was increased in maximum diameter (b) from 16 mm to 22 mm. (c) Coronal reconstruction shows periampullary duodenal diverticulum with a cranial ventral growth/expansion.

PAD may appear as rounded collection of gas material situated along the medial wall of the junction of the second and third portions of the duodenum on unenhanced CT, sometimes filled with fluid and misdiagnosed as a pancreatic abscess, pancreatic pseudocyst, or as a metastatic lymph node.^[4]^ For this reason, PAD is better characterized after oral and/or intravenous contrast material administration: oral contrast (about 800 mL of water or barium sulfate suspension of 2.1% weight/volume or diluted 2% water-soluble contrast material, approximately 1 h before scanning) could be helpful in evaluation of diverticulum size before and after administration, as diverticulum could double in maximum diameter after oral contrast and could reveal underlying extrinsic compression on biliary duct [Figure 2]. Intravenous contrast injection could be necessary in differential diagnosis between PAD and biliopancreatic neoplasm, abscess, or pseudocyst. Diverticulum walls demonstrate weak homogeneous enhancement, greatest in venous phase at about 45 s after injection, with no mass-like or washout behavior. Moreover, intravenous contrast agent highlights common bile duct walls and papilla of Vater, which are often compressed in Lemmel syndrome. CT is also used to find out the most common causes of biliopancreatic disease, in particular, when US is inconclusive.

## CASE REPORTS

### Case 1

**Figure 3:**
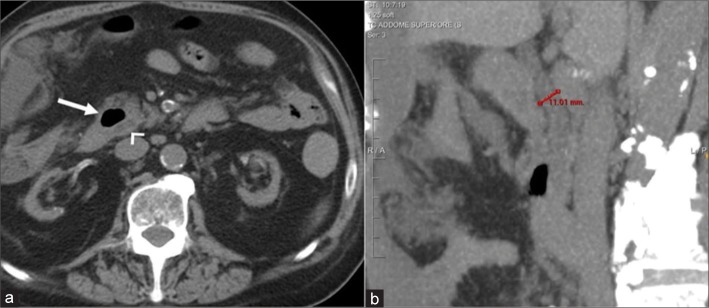
A 79-year-old man presented with epigastric pain. Unenhanced computed tomography scan of the abdomen demonstrated a 27-mm periampullary duodenal diverticulum filled with air (arrow in a) strictly adjacent to the distal portion of the common bile duct (arrowhead in a) which is 11 mm in maximum diameter (b). Symptoms regressed after NG decompression.

A 79-year-old male with acute abdominal pain after having presented several self-limiting episodes of abdominal pain in the right hypochondrium and epigastrium, underwent US examination in the emergency department, with no significative findings. He was discharged 6 h later. After 2 days, he was readmitted to the emergency department for increased epigastric pain happened about an hour after lunch. As slight increase in total bilirubin was found in blood test, unenhanced CT scan of the abdomen was required. It demonstrated the presence of a PAD associated with common bile duct dilatation [Figure 3]. No other causes of epigastric pain were found. A nasogastric tube was placed for decompression and suction. Follow-up laboratory values revealed normalization of the previously elevated total bilirubin.

### Case 2

A 70-year-old female presenting with nausea, vomiting, weight loss, and slight jaundice was evaluated for acute cholecystitis. Laboratory values revealed that hemoglobin was normal, but bilirubin metabolites and inflammatory markers, including CRP, were elevated. US demonstrated dilated common bile duct with no signs referable to lithiasis. A contrast-enhanced CT scan was required to study in deep pancreatic head region.

**Figure 4:**
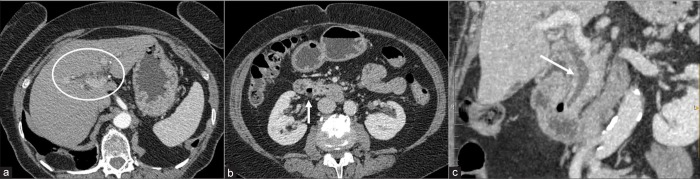
Contrast-enhanced computed tomography in a 70-year-old woman with nausea, vomiting, and slight jaundice. Intrahepatic bile duct dilatation is shown in a (white circle) with a small periampullary duodenal diverticulum with airfluid level demonstrated in b (arrowhead). Coronal reconstruction (c) highlights common bile duct dilatation and its close relation with periampullary duodenal diverticulum (arrow).

CT retrospectively revealed the presence of a 17-mm PAD [Figure 4] and confirmed intra- and extra-hepatic biliary tract dilatation (common bile duct of 13 mm). She was treated with 7-day course of intravenous wide spectrum antibiotics with no recurrence of symptoms within 3 months.

## DISCUSSION

We reported two cases of PAD related to Lemmel syndrome. Lemmel syndrome is an unrecognized cause of jaundice and/or common bile duct dilatation because more frequent and important causes (choledocholithiasis or pancreaticobiliary and periampullary tumors) are first depicted. Imaging is essential to correctly identify and diagnose Lemmel syndrome, as awareness of this condition may prevent mismanagement. CT scan is the reference imaging modality because majority of patients present to emergency department with acute abdominal pain. US could reveal biliary duct dilatation, but the inherent limited ability to examine bowel pathology results in non-depiction of the duodenal diverticula. PAD could be evaluated through CT, MRI, or ERCP, which could be also therapeutic.

There are few data reported in literature about imaging related to Lemmel syndrome, mainly case reports evaluated with ERCP.^[3,5-7]^

Kouraklis *et al.*^[8]^ in their study on small bowel diverticular disease described three cases of symptomatic duodenal diverticula focusing on the clinical findings without imaging correlation. Abdominal cross-sectional imaging has been reported to be a useful diagnostic tool in defining duodenal pathology and overcoming the aforementioned difficulties of clinical and ultrasound examinations.^[5]^

A study of Perdikakis *et al.*^[3]^ reported 10 patients, all presenting with acute abdomen and diagnosed with duodenal diverticula, documented with MDCT in eight cases, in which they focused on the importance of both axial and coronal plane in demonstrating the presence of the diverticula.

Rouet *et al.*^[8]^ presented a case of Lemmel’s syndrome with a large PAD causing obstructive jaundice and mimicking periampullary tumor: in this case, an endoscopic ultrasound was performed, revealing a 2 cm diameter duodenal diverticulum with a small flange that increased in size (more than 50%) on instillation of water and resulted in obvious compression of both the biliary tract and main pancreatic duct, confirming the diagnosis of Lemmel’s syndrome. We also demonstrated that after oral contrast administration, PAD could significative increase in size, causing dilatation of common bile duct, even if oral hyperdense contrast media could hinder small CBD stones.

PAD is best demonstrated using a side-viewing endoscope, but it remains an invasive imaging modality which could reveal that the distal portion of the common bile duct is laterally compressed by the PAD.

Patients who do not undergo ERCP or endoscopic examination may benefit from the ability of MR and magnetic resonance cholangiopancreatography (MRCP) to detect PAD, enabling determination of the etiology of clinical symptoms and guidance for appropriate management.^[5]^

Although duodenal diverticula can be found in up to 22% of the population, only 1–2% of these patients become symptomatic.^[9,10]^ Conservative management consists of nasogastric decompression and wide spectrum antibiotic coverage in cases of perforation. Therefore, duodenal diverticula are usually untreated unless complications occur. If conservative treatment options fail, surgical transduodenal diverticulectomy is usually performed.

## CONCLUSION

Although duodenal diverticula constitute a rare cause of acute abdomen, careful imaging analysis can aid to the identification of this uncommon factor of abdominal symptomatology. Contrast-enhanced CT is the imaging of choice for a rapid, non-invasive, and specific evaluation of PAD associated with Lemmel syndrome. Oral contrast could help in confirming size-related symptoms.

## References

[1] Lemmel G. (1934). The clinical significance of the duodenal diverticulum. (Die klinische bedeutung der duodenaldivertikel) (Article in German). Arch Dig Dis.

[2] Egawa N, Anjiki H, Takuma K, Kamisawa T (2010). Juxtapapillary duodenal diverticula and pancreatobiliary disease. Dig Surg.

[3] Perdikakis E, Chryssou EG, Karantanas A (2011). Diagnosis of periampullary duodenal diverticula: The value of new imaging techniques. Ann Gastroenterol.

[4] McCullough KM. (1991). Duodenal diverticulum simulating a pancreatic mass on computed tomography. Australas Radiol.

[5] Leivonen MK, Halttunen JA, Kivilaakso EO (1996). Duodenal diverticulum at endoscopic retrograde cholangiopancreatography, analysis of 123 patients. Hepatogastroenterology.

[6] Rouet J, Gaujoux S, Ronot M, Palazzo M, Cauchy F, Vilgrain V (2012). Lemmel’s syndrome as a rare cause of obstructive jaundice. Clin Res Hepatol Gastroenterol.

[7] Chiang TH, Lee YC, Chiu HM, Huang SP, Lin JT, Wang HP (2006). Endoscopic therapeutics for patients with cholangitis caused by the juxtapapillary duodenal diverticulum. Hepatogastroenterology.

[8] Kouraklis G, Glinavou A, Mantas D, Kouskos E, Karatzas G (2002). Clinical implications of small bowel diverticula. Isr Med Assoc J.

[9] Agundez MC, Guerra DL, Perez JF, Fernandez GB (2017). Lemmel’s syndrome: Obstructive jaundice secondary to a duodenal diverticulum. Cir Esp.

[10] Bergman S, Koumanis J, Stein LA, Barkun JS, Paraskevas S (2005). Duodenal diverticulum with retroperitoneal perforation. Can J Surg.

